# Interplay of Mediterranean-diet adherence, genetic factors, and metabolic dysfunction-associated steatotic liver disease risk in Korea

**DOI:** 10.1186/s12967-024-05408-z

**Published:** 2024-06-25

**Authors:** Yu-Jin Kwon, Ja-Eun Choi, Kyung-Won Hong, Ji-Won Lee

**Affiliations:** 1 Department of Family Medicine, Yongin Severance Hospital, Yonsei University College of Medicine, 363, Dongbaekjukjeon-daero, Giheung-gu, Yongin-si, Gyeonggi-do 16995 Korea; 2 Institute of Advanced Technology, Theragen Health Co. Ltd., Pangyoyeok-ro, Seongnam-si, Gyeonggi-do 13493 Republic of Korea; 3 Department of Family Medicine, Yonsei University College of Medicine, Severance Hospital, Yonsei-ro 50-1, Seodaemun-gu, Seoul, 03722 Republic of Korea; 4 Institute for Innovation in Digital Healthcare, Yonsei University, Yonsei-ro 50-1, Seodaemun-gu, Seoul, 03722, Republic of Korea

**Keywords:** Mediterranean diet, Metabolic dysfunction-associated fatty liver disease, Gene-diet interaction, Glucokinase regulatory protein gene

## Abstract

**Backgrounds:**

Metabolic dysfunction-associated steatotic liver disease (MASLD) has gained attention owing to its severe complications. This study aimed to explore the interaction between Mediterranean-diet (MD) adherence, genetic factors, and MASLD risk in a Korean population.

**Methods:**

In total, 33,133 individuals aged 40 years and older from the Korean Genome and Epidemiology Study (KoGES) were analyzed. Participants were assessed for MASLD based on criteria and MD adherence measured by the Korean version of the Mediterranean-Diet Adherence Screener (K-MEDAS). Individuals were categorized into two groups based on their MD adherence: high adherence (K-MEDAS > 6) and low adherence (K-MEDAS < 5). Single nucleotide polymorphism (SNP) genotypes were obtained using the Korea Biobank array. Logistic regression was used to examine the single-marker variants for genetic associations with MASLD prevalence.

**Results:**

Individuals were categorized into MASLD (10,018 [30.2%]) and non-MASLD (23,115 [69.8%]) groups. A significant interaction was observed between the rs780094 glucokinase regulatory protein (GCKR) gene and K-MEDAS on MASLD (p < ${10}^{-2}$). Of individuals with K-MEDAS > 6, those carrying the minor allele (C) of the GCKR gene rs780094 exhibited a lower risk of MASLD compared to those without the allele (odds ratio [OR] = 0.88 [0.85–0.91], p-value = 5.54e^−13^).

**Conclusion:**

The study identified a significant interaction involving the rs780094 variant near the GCKR gene, with carriers of the minor allele exhibiting a lower MASLD risk among those adhering well to the MD. Dietary habits influence the MASLD risk associated with the rs780094 allele, emphasizing the need for personalized nutrition recommendations.

**Supplementary Information:**

The online version contains supplementary material available at 10.1186/s12967-024-05408-z.

## Introduction

Over the years, the prevalence of metabolic disorders, such as obesity, metabolic syndrome, and diabetes, has significantly increased in South Korea and globally [1, 2]. This phenomenon results from complex interactions between eating habits, industrialization, changes in social environments, and genetic factors [3]. In particular, the rising incidence of metabolic abnormalities, such as nonalcoholic fatty liver diseases (NAFLD), has raised significant concerns among medical and public health authorities [4]. Recently, the metabolic dysfunction-associated steatotic liver disease (MASLD) concept, which focuses on abnormalities in metabolic function playing a significant role in its etiology, has gained wide acceptance [5]. MASLD is closely associated with metabolic disorders, and it can progress to liver-related complications such as cirrhosis, liver failure, hepatocellular carcinoma, and death [6]. However, the complex pathogenic mechanism underlying the onset and progression of MASLD remains unclear. Recent research has suggested that the complex interplay between genetic variations, lifestyles, and dietary habits may influence the occurrence and progression of MASLD [7].

The Mediterranean diet (MD), a dietary pattern that originates from the Mediterranean region, such as Greece, Spain, and Italy, is characterized by a high intake of vegetables, legumes, fruits, nuts, cereals, and olive oil, with low saturated fat intake and moderate-to-high fish consumption [8]. The MD is well known for preventing heart disease, diabetes, and mortality [8–11]. Several meta-analyses have established that greater adherence to the MD enhances the primary prevention of major chronic diseases, including a significant reduction in mortality, cardiovascular disease, and diabetes [12, 13]. However, these benefits may manifest differently in various population groups with diverse genetic backgrounds and dietary cultures. Therefore, our previous study developed a new MD-adherence questionnaire called the Korean version of the Mediterranean-Diet Adherence Screener (K-MEDAS), which is tailored to the dietary habits of Koreans [14]. Given the large-scale epidemiological nature of this study, we employed K-MEDAS, which is based on a Food Frequency Questionnaire (FFQ), to assess MD adherence among Korean participants. Based on these processes, this study aimed to investigate the role of genetic variations and MD adherence in the prevalence of MASLD using a large prospective cohort of middle-aged Korean adults. To our knowledge, this is the first study to investigate the interplay between MD adherence, MASLD, and genetic factors in the Korean population.

## Methods

### Study population

This cross-sectional study examines the interplay of genetic and MD diet in prevalent MASLD. This study utilized data from population-based cohorts within the Korean Genome and Epidemiology Study (KoGES), which includes the KoGES_Ansan and Ansung study (a community-based cohort in urban and rural counties), the KoGES_health examinee study (a national health examinee registry), and the KoGES_cardiovascular disease association study (a community-based cohort in rural counties). In the KoGES_Ansan and Ansung study, participants aged 40–69 years voluntarily enrolled at baseline between 2001 and 2002. For the KoGES_health examinee study, participants aged 40 years and over were enrolled at baseline from 2004 to 2013. In the KoGES_cardiovascular disease association study, participants aged 40–69 years were enrolled at baseline from 2005 to 2011.

For baseline recruitment, eligible participants were invited to participate through various methods such as on-site invitations, mailed letters, telephone calls, media campaigns, or community leader-mediated conferences. Those who expressed interest were asked to visit survey sites, comprising more than 50 national and international medical schools, hospitals, and health institutions. At these sites, participants underwent interviews, completed questionnaires administered by trained staff, and underwent physical examinations. The primary reasons for declining participation included changes in contact information (telephone numbers or mailing addresses), being too busy to attend, and not responding to telephone calls [15].

Within the KoGES, 72,299 individuals aged 40 years and older who have genome-wide single nucleotide polymorphism (SNP) genotype data were found. Participants within KoGES who had been diagnosed with liver diseases (n = 374), consumed alcohol > 210 g/week in men and 140 g/week in women (n = 6.283), had missing data pertaining to laboratory or anthropometric measurements (n = 4.778) were excluded. To distinctly investigate the genetic variations based on MD adherence, the participants were stratified into tertile groups based on the Korean version of the Mediterranean-Diet Adherence Screener (K-MEDAS) (T1: < 5; n = 13,154, T2: 5 and 6; n = 27,731, T3: > 6, n = 19,979). Subsequently, Genome-Wide Association Studies (GWAS) analysis was exclusively conducted on the upper (top 33.3%) and lower (bottom 33.3%) tertile groups. Therefore, individuals with a K-MEDAS score of 5 or 6 (n = 27,731) were also excluded. In total, 33,133 participants were included in the final analysis. Within the lower tertile group (K-MEDAS < 5; n = 13,154) a comparison of SNP differences was conducted between the without (n = 8.631) and MASLD (n = 4.523) participants. Similarly, within the upper tertile group (K-MEDAS > 6; n = 19,979), an analysis of SNP differences was performed between the individuals without (n = 14,484) and with MASLD (n = 5,495).

Informed consent was provided and obtained from all participants in this study. This study was approved by the Institutional Review Board, Theragen Bio (approval number: 700062-220814-GP-007-003).

### Assessment of dietary intake and MD adherence

For dietary assessment, a semi-quantitative FFQ was designed, incorporating 103 items, and administered as part of KoGES [16]. Respondents reported the consumption frequency and portion sizes of foods consumed over the preceding year. The analysis of questionnaire data, referencing a food composition database, facilitated the estimation of dietary intakes. Based on the FFQ data, total calories, macronutrient intakes, and micronutrient intakes were estimated.

The K-MEDAS questionnaire, previously developed and validated in a separate study, was employed to assess adherence to the Mediterranean Diet (MD) [14]. Consisting of 14 questions, each scored as either 0 or 1, the K-MEDAS questionnaire yielded a total score ranging from 0 to 14 points. Based on FFQ, foods were categorized to derive a corresponding score for the K-MEDAS. A higher score indicated stronger adherence to the MD. Detailed information about the K-MEDAS questionnaire is provided in the Table S1.

### Covariates

Body mass index (BMI) was computed as weight (kg) divided by the square of height (m). Waist circumference (WC) was measured at the midpoint between the lowest rib and the iliac crest. Blood pressures were measured twice using a mercury sphygmomanometer while sitting. Smoking status was classified into three categories: non-smokers, ex-smokers, and current smokers. Alcohol consumption was categorized as non-drinkers (consuming alcohol fewer than 12 times annually, with a single serving not exceeding one cup), ex-drinkers, and current drinkers. Regular exercise leading to perspiration was considered indicative of engagement in physical activity. Participants were requested to fast for at least 8 h before undergoing blood tests. Total cholesterol (TC), triglyceride (TG), high-density lipoprotein (HDL) cholesterol, blood glucose, glycated hemoglobin (HbA1c), insulin, C-reactive protein (CRP), γ-glutamyltransferase (γ-GGT), aspartate transaminase (AST), and alanine transaminase (ALT) were measured by enzymatic methods using a chemistry analyzer (Hitachi 7600, Tokyo, Japan by August 2002 and ADVIA 1650, Siemens, Tarrytown, NY from September 2002) according to standardized protocol [15]. Homeostasis model assessment of insulin resistance (HOMA-IR) was calculated as follows: HOMA-IR = (fasting plasma glucose (FPG) in mmol/L × fasting serum insulin in pmol/L) ÷ 22.5 [17]. Hypertension was defined as either a systolic (SBP) or a diastolic blood pressure (DBP) of ≥ 140 mmHg or ≥ 90 mmHg, respectively, or when participants self-reported the diagnosed diseases [18]. Type 2 diabetes was defined as either a fasting blood glucose level of ≥ 126 mg/dL or HbA1c ≥ 6.5% or when participants self-reported the diagnosed diseases [19]. Overweight and obese were defined as BMI ≥ 23 kg/m^2^ and ≥ 25 kg/m^2^, respectively [20].

### Assessment of MASLD

A positive diagnosis of MASLD was established based on the presence of blood biomarker evidence of hepatic steatosis and meeting one of the following three criteria: overweight or obesity, the presence of type 2 diabetes, or the presence of at least two metabolic risk abnormalities [21]. Metabolic risk abnormalities are as follows: (1) WC ≥ 90 cm in men and WC ≥ 80 cm in women, (2) SBP ≥ 130 mmHg or DBP ≥ 85 mmHg or drug treatment, (3) TG ≥ 150 mg/dL or drug treatment, (4) HDL-C < 40 mg/dL in men and < 50 mg/dL in women, (5) presence of prediabetes (fasting glucose level 100–125 mg/dL or HbA1c 5.7–6.4%), (6) HOMA-IR score ≥ 2.5, and (7) CRP > 2 mg/dL.

The formula for calculating the Fatty Liver Index (FLI) score is as follows:

FLI = [e^(0.953 × ln(TGs) + 0.139 × BMI + 0.718 × ln(γGTP) + 0.053 × WC − 15.745)] / [1 + e^(0.953 × ln(TGs) + 0.139 × BMI + 0.718 × ln(γGTP) + 0.053 × WC − 15.745)] × 100 [22, 23]. The presence of hepatic steatosis was defined as an FLI > 30 [22, 23].

### Genotyping

Fasting blood samples were collected in a one-serum separator tube and two ethylenediaminetetraacetic acid tubes. Blood DNA samples were prepared and transported to the National Biobank of Korea. SNP genotypes were obtained using the Korea Biobank array (KoreanChip), which was specifically designed for the Korean population to facilitate GWAS on blood biochemical traits. The KoreanChip included > 833,000 markers, encompassing over 247,000 rare or functional variants, derived from the sequencing data of over 2,500 Koreans [24]. Detailed information about the KoreanChip can be found in a previous study [24]. Rigorous criteria were applied during KoreanChip data analysis to ensure genotyping quality: a call rate of > 97%, a missing genotype rate of < 0.01, a minor allele frequency of > 0.01, and a Hardy–Weinberg equilibrium p-value of > 0.000001.

### Statistical analysis

Data were presented as mean ± standard deviation or as number (percentages). Continuous variables were evaluated using students’ t-tests to compare individuals with and without MASLD, while categorical variables were assessed using Chi-squared tests. Principal component analysis was implemented to mitigate genomic data bias stemming from sample collection regions. Principal component (PC) 1 and PC 2 were included as covariates in subsequent statistical analyses. Logistic regression was applied to examine the single-marker variants for genetic associations with MASLD prevalence, with adjustments for age, sex, BMI, exercise, alcohol, smoking, PC1, and PC2. All statistical analyses were carried out using PLINK software (version 1.07). Significance was determined at p < 5 × 10^–8^. Values of p < 5 × 10^–2^ were considered statistically significant for the gene-MD interaction.

## Results

Table 1 presents the clinical characteristics of the study population with or without MASLD. Of the total 33,133 study participants, 10,018 (30.2%) had MASLD, while 23,115 (69.8%) did not. Participants with MASLD were more likely to be male and older, with higher levels of BMI, WC, SBP, DBP, fasting glucose, HbA1c, insulin, CRP, HOMA-IR, TC, TG, γ-GGT, AST, ALT, and lower levels of HDL-C. Furthermore, participants with MASLD had a higher proportion of current smokers, current alcohol drinkers, hypertension, and type 2 diabetes, while a lower proportion engaged in regular exercise. Regarding nutritional status, participants with MASLD had a higher total calorie intake and higher intake levels of carbohydrates (g), proteins (g), niacin (mg), zinc (μg), and sodium (mg). Additionally, they had lower intake levels of calcium (mg), iron (mg), potassium (mg), vitamin C (mg), folate (μg), fiber (g), vitamin E (mg), and cholesterol (mg). The mean K-MEDAS score was significantly higher in participants without MASLD compared to those with MASLD.

**Table 1:** Clinical characteristics of the study population

Variables	Total	Without MASLD	With MASLD	p-value
N	33,133	23,115	10,018	
Age	54.3 ± 8.3	53.7 ± 8.2	55.7 ± 8.2	< 0.001
Female, n (%)	23,307 (70.2)	18,233 (78.9)	5061(50.5)	< 0.001
BMI, kg/m^2^	23.9 ± 2.9	22.8 ± 2.2	26.6 ± 2.6	< 0.001
WC, cm	80.7 ± 8.6	77.1 ± 6.8	89.0 ± 6.5	< 0.001
SBP, mmHg	121.9 ± 15.5	119.7 ± 15.1	127.1 ± 15.2	< 0.001
DBP, mmHg	75.5 ± 10.0	74.0 ± 9.7	78.9 ± 9.9	< 0.001
FPG, mg/dL	94.7 ± 19.4	92.1 ± 16.2	100.6 ± 24.3	< 0.001
HbA1c, %	5.7 ± 0.7	5.6 ± 0.6	6.0 ± 0.9	< 0.001
Insulin, IU	7.9 ± 4.2	7.1 ± 3.9	9.0 ± 4.3	< 0.001
HOMA-IR	1.8 ± 1.2	1.6 ± 0.9	2.2 ± 1.5	< 0.001
Hs CRP, mg/dL	0.33 ± 1.63	0.25 ± 1.33	0.53 ± 2.2	< 0.001
TC, mg/dL	197.8 ± 35.9	195.1 ± 34.6	204.1 ± 38.0	< 0.001
HDL-C, mg/dL	52.7 ± 13.1	55.5 ± 13.1	46.1 ± 10.6	< 0.001
TG, mg/dL	125.2 ± 82.4	98.0 ± 46.2	187.6 ± 109.1	< 0.001
γ-GGT, IU/L	26.7 ± 29.5	19.2 ± 13.6	43.4 ± 43.5	< 0.001
AST, IU/L	23.7 ± 15.2	22.2 ± 8.2	27.2 ± 24.2	< 0.001
ALT, IU/L	22.2 ± 19.0	18.8 ± 12.4	30.2 ± 27.3	< 0.001
Current drinking, n (%)	12,510 (37.9)	7.976 (34.7)	4.478 (45.0)	< 0.001
Current smoking, n (%)	3.029 (9.2)	1.432 (6.2)	1.570 (15.8)	< 0.001
Physical activity, n (%)	17,196 (52.0)	12,323 (53.5)	4.845 (45.6)	< 0.001
Hypertension, n (%)	6.745 (20.3)	3.564 (15.4)	3.175 (31.7)	< 0.001
Type2 diabetes, n (%)	2.283 (6.9)	1.210 (5.2)	1.073 (10.7)	< 0.001
Dyslipidemia, n (%)	3.409 (10.3)	1.952 (8.5)	1.454 (14.5)	< 0.001
Mediterranean diet score	6.0 ± 2.2	6.1 ± 2.2	5.8 ± 2.1	< 0.001
Energy, kcal	1746.1 ± 570.2	1733.0 ± 570.6	1776.3 ± 568.3	< 0.001
Protein, g	58.9 ± 26.1	58.6 ± 25.9	59.4 ± 26.8	< 0.001
Fat, g	27.2 ± 17.8	27.2 ± 17.6	27.3 ± 18.3	0.618
Carbohydrate, g	312.5 ± 94.3	309.9 ± 94.9	318.6 ± 92.8	< 0.001
Protein, %	13.3 ± 2.6	13.2 ± 2.6	13.4 ± 2.6	< 0.001
Fat, %	13.5 ± 5.4	13.3 ± 5.4	13.6 ± 5.4	< 0.001
Carbohydrate, %	72.2 ± 6.9	72.4 ± 6.9	72.1 ± 6.9	< 0.001
Calcium, mg	460.2 ± 279.7	467.9 ± 283.2	442.4 ± 270.7	< 0.001
Phosphorus, mg	900.7 ± 375.5	900.7 ± 376.3	900.9 ± 373.6	0.963
Iron, mg	10.2 ± 5.4	10.2 ± 5.4	10.0 ± 5.2	< 0.001
Potassium, mg	2283.2 ± 1129.9	2295.7 ± 1138.1	2255.4 ± 1111.0	< 0.001
Sodium, mg	2521.2 ± 1458.9	2480.4 ± 1423.1	487.3 ± 370.5	< 0.001
Vitamin A, R. E	490.4 ± 368.7	491.8 ± 367.7	2615.5 ± 1533.8	0.317
Vitamin B1, mg	1.00 ± 0.46	1.00 ± 0.45	1.02 ± 0.47	< 0.001
Vitamin B2, mg	0.90 ± 0.46	0.91 ± 0.46	0.90 ± 0.46	0.043
Niacin, mg	14.4 ± 6.3	14.3 ± 6.3	14.6 ± 6.4	< 0.001
Vitamin C, mg	111.4 ± 75.8	113.4 ± 77.0	106.9 ± 73.0	< 0.001
Zinc, μg	7.9 ± 3.7	7.9 ± 3.6	8.0 ± 3.9	0.012
Vitamin B6, mg	1.60 ± 0.73	1.60 ± 0.73	1.60 ± 0.74	0.706
Folate, μg	223.9 ± 131.1	225.6 ± 131.9	220.0 ± 129.3	< 0.001
Fiber, g	6.0 ± 3.2	6.0 ± 3.2	5.9 ± 3.1	0.026
Vitamin E, mg	8.3 ± 4.8	8.3 ± 4.8	8.2 ± 4.7	0.037
Cholesterol, mg	166.9 ± 127.5	169.2 ± 127.7	161.8 ± 127.0	< 0.001

Data are presented as mean ± standard deviations or number (%)

The independent t-test was used to compare continuous variables, while the chi-square test was used to compare categorical variables

The percentages of energy from carbohydrates, sugars, and proteins were calculated as follows: carbohydrates (g), sugar (g), or protein (g) × 4 kcal/total energy intake (kcal) × 100. The percentages of energy from fatty acids were calculated as follows: fat (g) × 9 kcal/total energy intake (kcal) × 100

*MASLD* Metabolic dysfunction-associated steatotic liver disease, *BMI* Body mass index, *WC* Waist circumference, *FPG* Fating plasma glucose, *HbA1c* Glycated hemoglobin, *HOMA-IR* Homeostatic Model Assessment for Insulin Resistance, *hsCRP* High-sensitivity C-reactive protein, *TC* Total cholesterol, *HDL-C* High-density lipoprotein cholesterol, *TG* Triglyceride, *r-GGT* r-Glutamyltransferase, *AST* Aspartate transaminase, *ALT* Alanine transaminase

**Fig. 1:**
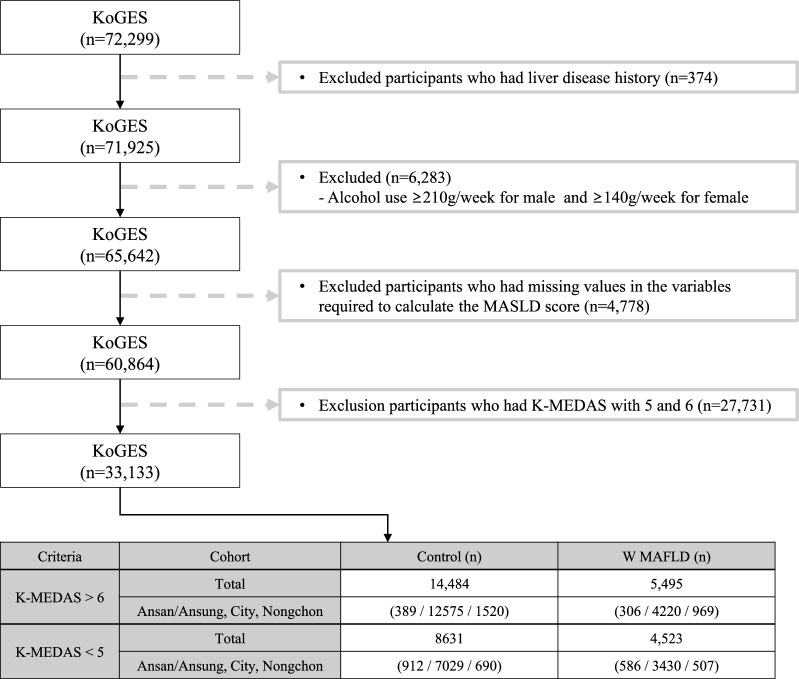
Flow chart of study population. *KoGES* Korean Genome and Epidemiology Study, *MASLD* Metabolic dysfunction-associated steatotic liver disease, *K-MEDAS* Korean version of the Mediterranean-Diet Adherence Screener. In total, 33,133 participants (K-MEDAS < 5; n = 13,154 and K-MEDAS > 6; n = 19,979) were included in the final analysis. Within the lower tertile group (K-MEDAS < 5; n = 13,154), a comparison of SNP differences was conducted between participants without (n = 8.631) and with MASLD (n = 4,523). Within the upper tertile group (K-MEDAS > 6; n = 19,979), an analysis of SNP differences was performed between individuals without (n = 14,484) and with MASLD (n = 5.495)

Table S2 presents the clinical characteristics of the study population without MASLD, categorized by K-MEDAS scores > 6 or < 5. The presentation includes data before and after propensity score matching, with consideration given to age and sex. Of 23,115 study participants, 14,484 (62.6%) had K-MEDAS scores > 6, while 8,631 (37.3%) had K-MEDAS scores < 5. Following propensity score matching, 446 participants each had K-MEDAS scores > 6 and < 5. Table S3 presents the clinical characteristics of the study population with MASLD, categorized by K-MEDAS scores > 6 or < 5. The presentation also includes data before and after propensity score matching, with consideration given to age and sex. Following propensity score matching, 415 participants each had K-MEDAS scores > 6 and < 5 (Fig. 1).

**Fig. 2:**
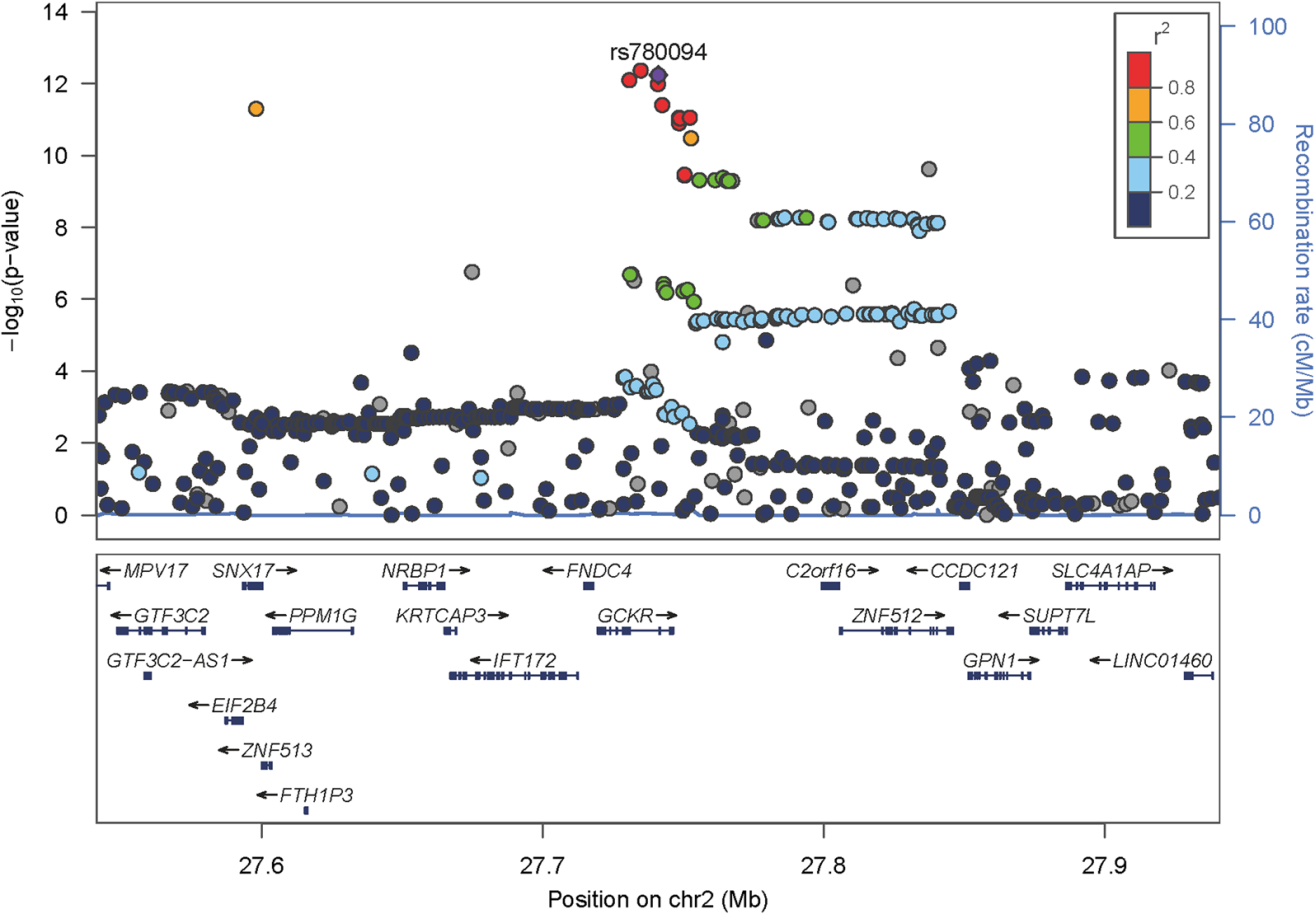
Regional association plots of the rs780094

Table 2 shows the significant SNP with significant association (p < ${10}^{-8}$) with MASLD and with significant gene-MD interaction (p < ${10}^{-2}$). Of individuals with K-MEDAS > 6, those carrying the minor allele (C) of the glucokinase regulatory protein (GCKR) gene rs780094 exhibited a lower risk of MASLD compared to those not carrying the allele (odds ratio [OR] = 0.88 [0.85–0.91], p-value = 5.54e^−13^). However, in individuals with K-MEDAS < 5, a weaker association was observed between rs780094 and MASLD (OR = 0.93 [0.88–0.98], p-value = 5.72e^−03^). Regional association plots of the rs780094 are illustrated in Fig. 2.

**Table 2:** Single nucleotide polymorphisms (SNPs) showing significant interactions with K-MEDAS associated with MASLD

SNP	CHR	BP	A1	Gene	ALT	REF	1000 G frequency		
							EAS	EUR	AMR
rs780094	2	27741237	C	GCKR	C	T	0.5238	0.5895	0.6398
	Total		K-MEDAS > 6		K-MEDAS < 5		Genotype x K-MEDAS		
SNP	OR (95% CI)	p-value	OR (95% CI)	p-value	OR (95% CI)	p-value	OR (95% CI)	p-value	
rs780094	0.88 (0.85–0.91)	5.5e^−13^	0.84 (0.80–0.88)	5.8e^−13^	0.93 (0.88–0.98)	5.7e^−03^	0.91 (0.84–0.99)	1.9^e−02^	

*SNP* Single nucleotide polymorphisms, *K-MEDAS* Korean version of the Mediterranean-Diet Adherence Screener, *MASLD* Metabolic dysfunction-associated steatotic liver disease, *Chr* chromosome, *BP* base pair, *EAS* East Asian, *EUR* European, *AMR* American, *N/A* not applicable, *MAF* major allele frequency, *A1* minor allele, *OR* odds ratio, *95% CI* 95% confidence interval

**Fig. 3:**
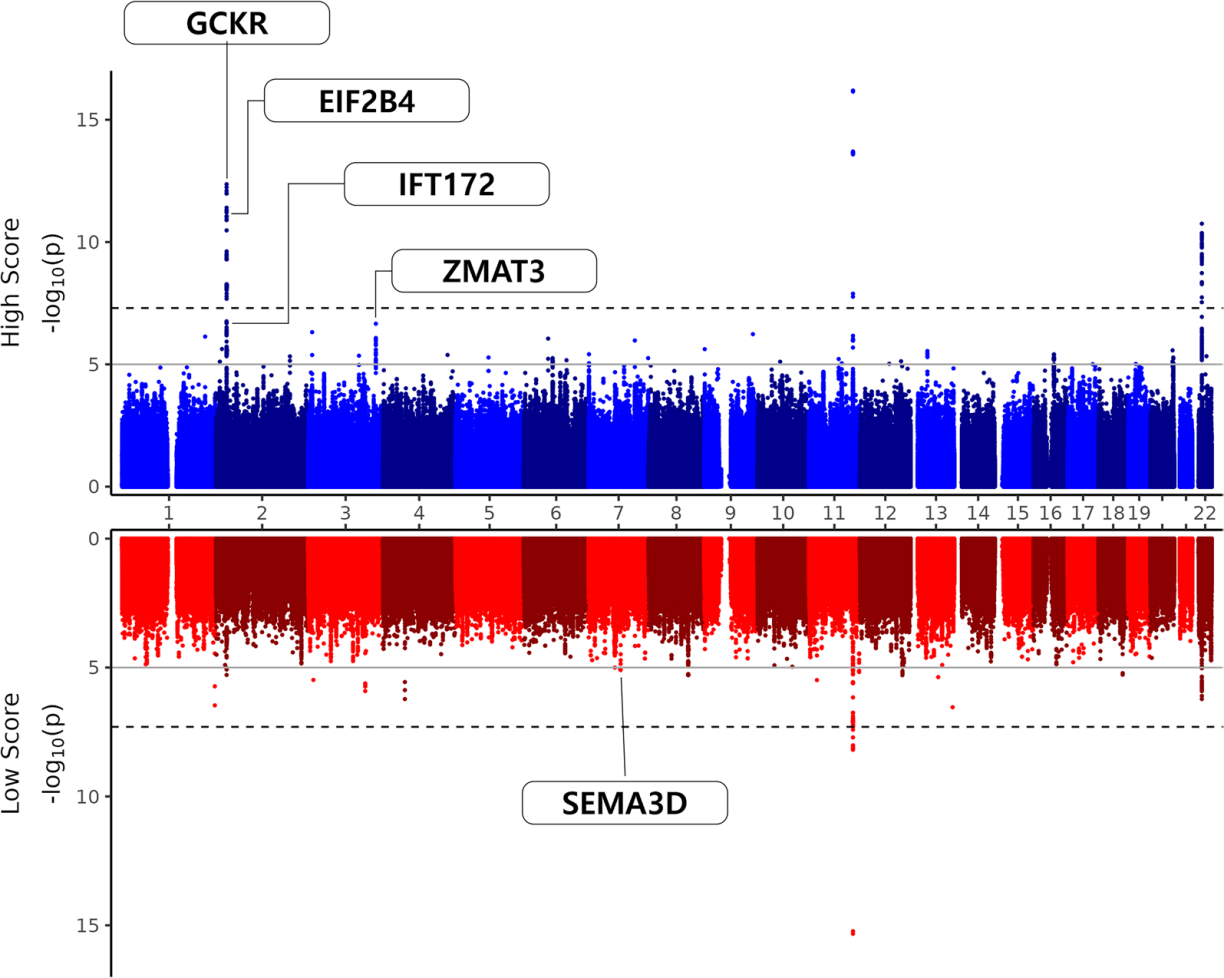
Miami plot showing P values for the SNP associations with MASLD in participants whose K-MEDAS was either < 5 or > 6

Supplementary Table 4 presents the SNPs with significant MD scores by gene interaction (p < ${10}^{-2}$) associated with MASLD. Forty gene-by-MD score interactions were associated with MASLD. Figure 3 presents a Miami plot showing p values for the SNP associations with MASLD in participants whose K-MEDAS was either < 5 or > 6. We observed genome-wide significant association clusters of GCKR, EIF2B4, IFT172, and ZMAT3 participants with K-MEDAS > 6, and for SEMA3D in participants with K-MEDAS < 5.

We conducted a subgroup analysis based on sex (Table S5). For males, among individuals with a K-MEDAS score greater than 6, those carrying the minor allele (C) of the GCKR gene rs780094 exhibited a lower risk of MASLD compared to those not carrying the allele (OR = 0.80 (0.74–0.88), p-value = 4.4e^−07^). Although the p-value was not statistically significant, it indicated a considerable trend toward significance. For females, among individuals with a K-MEDAS score greater than 6, those carrying the minor allele (C) of the GCKR gene rs780094 also exhibited a lower risk of MASLD compared to those not carrying the allele (OR = 0.86 (0.81–0.91), p-value = 6.7e^−08^), which was statistically significant. However, in both males and females with a K-MEDAS score below 5, the statistical significance diminished (OR = 0.93 (0.86–1.01), p-value = 6.9e^−02^ for male and OR = 0.92 (0.85–0.99), p-value = 3.1e^−02^ for female).

## Discussion

In this study, we conducted a comprehensive investigation of the interaction between MD adherence, MASLD, and genetic factors within the Korean population. Notably, we identified a significant interaction involving a specific genetic variant, rs780094, located near the GCKR gene region, related to MASLD. We also introduced K-MEDAS as a tool for evaluating MD adherence in the Korean population. Of individuals with a high MD adherence (K-MEDAS > 6), carriers of the rs780094 minor allele exhibited a 12% lower risk of MASLD compared to those not carrying the SNP (p-value = 5.80E^−13^). However, among individuals with low MD adherence (K-MEDAS < 5), carriers of the rs780094 minor allele showed a 7% decreased risk of MASLD compared to those not carrying the SNP, though statistical significance was not observed (p-value = 5.72E^−03^). This suggests that certain genetic factors may modulate the impact of dietary choices on liver health.

The rs780094 variant in the GCKR gene is linked to several health conditions, including type 2 diabetes [25], metabolic syndromes [26], dyslipidemia, and their related traits [27]. Notably, several studies have identified the association between GCKR rs780094 and NAFLD [28, 29].

The GCKR gene, located on chromosome 2 in humans, encodes GCKR, which serves as a key regulator of GCK in the liver [30]. Functional GCKR gene variants affect glucokinase regulatory protein (GKRP) expression, localization, and sequestration ability, resulting in an easier dissociation of GK from GKRP and persistent stimulation of de novo lipogenesis [31]. Regulating GCK expression and activity is a highly intricate process that involves hormonal, metabolic, and environmental factors [32].

GCK, also known as hexokinase (HK) IV, plays a critical role in glucose homeostasis [32]. HK1–3 have high affinities for glucose, and the majority of glucose is processed by these HKs. While GCK has a lower affinity for glucose and is released from GKRP when an excessive amount of glucose enters the cell [32], GCK functions as a glucose sensor in pancreatic beta cells, playing a critical role in glucose-stimulated insulin secretion [33]. Hepatic GCK is modulated at both the transcriptional and post-translational levels [34]. Transcriptional regulation involves the control of GCK gene expression by insulin and glucagon, while post-translational regulation involves the inhibitory action of GCKR on GCK activity.

Two common GCKR variants (rs1260326 and rs780094) have opposing effects on glucose and TG metabolism [30, 35]. The study conducted by Saxena et al. [36] involved the analysis of 386,731 common SNPs in a cohort comprising 1,464 patients with type 2 diabetes and 1,467 matched controls. Their findings revealed that the T allele of the GCKR rs780094 variant is associated with lower levels of fasting glucose and insulin resistance and is correlated with higher levels of plasma TGs [36]. Orho-Melander et al. [35] confirmed that the GCKR rs780094 variant was linked to elevated plasma TG levels and lower levels of FPG. Additionally, they observed that the missense variant rs1260326 (C > T p.P446L), which showed strong linkage disequilibrium (r2 = 0.93) with rs780094, showed an association with plasma TG levels across 12 independent cohorts [35].

The relationship between the rs780094 variants in the GCKR gene and metabolic status is also influenced by specific dietary patterns [37, 38]. The pronounced interaction observed was between the consumption of whole-grain foods and rs780094 (in GCKR), particularly concerning fasting insulin concentrations [37]. Additionally, n-3 polyunsaturated fatty acids (PUFA) may play a contributing role in triggering insulin resistance and serum levels of CRP by interacting with a rs1260326-P446L genetic variant at GCKR [38].

In this study, of individuals with high MD adherence (K-MEDAS > 6), carriers of the rs780094 minor allele (C) showed a 12% lower risk of MASLD. Unlike previous studies focusing on different racial populations where the T allele was the minor allele, in our Korean study, the rs780094 C allele served as the minor allele. Notably, the overexpression of GKRP in diabetic rats led to enhanced glucose tolerance without triggering hepatic steatosis or elevated plasma TG levels [39]. This suggests the critical regulatory role of GKRP in maintaining a balance in hepatic glucose and lipid metabolism. Many assertions regarding gene-biomarker and gene-disease associations, as documented in published studies, have not been substantiated through replication when examined in independent populations.

In Pollin's study [40], another finding emerged. They did not observe a significant association between either the GCKR SNP or progression to diabetes in the Diabetes Prevention Program. Similarly, intensive lifestyle intervention seems to partially alleviate the impact of the 446L allele on elevated TG levels, while the P446 allele appears to amplify responsiveness to the HOMA-IR-lowering effect of metformin. In our study, of the participants adhering well to the MD, individuals with the rs780094 allele exhibited a lower risk of MASLD. Conversely, no statistical significance was observed in groups with low MD adherence. This suggests that lifestyle modifications may influence the risk of MASLD associated with the rs780094 allele, highlighting the impact of dietary habits on genetic predisposition.

Our study had some limitations. Firstly, it has a cross-sectional design, which precludes the establishment of causal relationships. Secondly, we calculated K-MEDAS based on the FFQ developed by the Korea Centers for Disease Control and Prevention. Moreover, variations in dietary cultures and food choices between Korea and Mediterranean coastal countries can influence the assessment of individuals' MD adherence. Notably, K-MEDAS is an adapted and validated version of an MD adherence screener originally designed for the Mediterranean region. Furthermore, it has been validated as a reliable tool for assessing MD adherence within the Korean population [14]. Third, we employed an FLI score for diagnosing NAFLD, which may not be as precise as histological confirmation or other advanced imaging modalities. However, in large-scale population-based epidemiological studies, using the FLI score is valuable, and previous research has demonstrated its good accuracy [41, 42]. Forth, blood sampling was conducted after a minimum fasting period of 8 h, which may not be as accurate as the recommended 10–12 h fasting period for lipid profile measurements. Additionally, this study analyzed secondary data from the cohort conducted by the Korea Disease Control and Prevention Agency, making it impossible to obtain specific information regarding the quality control material results. Finally, this study does not comprise a statistically random sample that is representative of the entire population, as is common in many prospective cohort studies. Therefore, it is important to consider these factors when applying the results to the entire population.

Despite these limitations, this study is the first to explore the interplay between MD adherence, genetic factors, and MASLD in a Korean population.

## Conclusion

The identified rs780094 gene, located near the GCKR gene region, modified the effects of the MD on the MASLD risk in the Korean population. The results underscore the potential benefits of an MD in reducing MASLD risk, with specific genetic markers modulating these effects. This research provides valuable insights into developing personalized nutrition recommendations and highlights the need for continued investigation into the complex pathogenic mechanisms of MASLD.

### Supplementary Information

Supplementary Material 1.

## Data Availability

Data in this study were from the Korean Genome and Epidemiology Study and are available on the following website: https://www.kdca.go.kr/contents.es?mid=a40504010000

## Abbreviations

ALT: Alanine transaminase
AST: Aspartate transaminase
BMI: Body mass index
CRP: C-reactive protein
DBP: Diastolic blood pressure
FPG: Fasting plasma glucose
FLI: Fatty liver index
FFQ: Food frequency questionnaire
GWAS: Genome-wide association studies
GCKR: Glucokinase regulatory protein
γ-GGT: γ-Glutamyltransferase
HbA1c: Glycated hemoglobin
HDL: High-density lipoprotein
HOMA-IR: Homeostasis model assessment insulin resistance
K-MEDAS: Korean version of the Mediterranean-diet adherence screener
MD: Mediterranean diet
KoGES: Korean genome and epidemiology study
MASLD: Metabolic dysfunction-associated fatty liver disease
NAFLD: Nonalcoholic fatty liver diseases
SNP: Single nucleotide polymorphism
SBP: Systolic blood pressure
TC: Total cholesterol
TG: Triglyceride
PUFA: Polyunsaturated fatty acids
WC: Waist circumference
