# Supplementary material for: Interplay of Mediterranean-diet adherence, genetic factors, and metabolic dysfunction-associated steatotic liver disease risk in Korea
Source: J Transl Med. 2024 Jun 25;22:591. doi: 10.1186/s12967-024-05408-z (PMC11197258; doi:10.1186/s12967-024-05408-z)
Supplement: Supplementary file 1 — Supplementary Material 1. [file 12967_2024_5408_MOESM1_ESM.docx]

**Supplementary materials**

**Interplay of Mediterranean-diet adherence, genetic factors, and metabolic dysfunction-associated steatotic liver disease risk in Korea**

**Short title: Genetic variation, Mediterranean diet, and MASLD**

Yu-Jin Kwon^1†^, Ja-Eun Choi^2†^, Kyung-Won Hong^2*^, Ji-Won Lee^3*^

^1^ Department of Family Medicine, Yongin Severance Hospital, Yonsei University College of Medicine, 363, Dongbaekjukjeon-daero, Giheung-gu, Yongin-si 16995, Gyeonggi-do, Korea

^2^R&D Division, Theragen Health Co. Ltd., Pangyoyeok-ro, Seongnam-si, Gyeonggi-do 13493, Republic of Korea

^3^ Department of Family Medicine, Yonsei University College of Medicine, Severance Hospital, Yonsei-ro 50-1, Seodaemun-gu, Seoul, Republic of Korea, 03722

†These co-first authors contributed equally to this work.

***Corresponding authors**

Ji-Won Lee, M.D., Ph.D.

Department of Family Medicine, Yonsei University College of Medicine, Severance Hospital, Yonsei-ro 50-1, Seodaemun-gu, Seoul, Republic of Korea, 03722

Tel: +82 2 2019 3480; Fax: +82 3462 8209; E-mail: [indi5645@yuhs.ac](mailto:indi5645@yuhs.ac)

Hong-Kyung Won, Ph.D.

R&D Division, Theragen Health Co. Ltd., Pangyoyeok-ro, Seongnam-si, Gyeonggi-do 13493, Republic of Korea

Tel: +82-10-3922-7089, Fax: +82-31-288-1294, E-mail: [kyungwon.hong@ theragenhealth.com](mailto:kyungwon.hong@theragenbio.com)

**Supplementary Table 1. Korean MEDAS questions data from the FFQ**

| Q1. Do you usually use perilla oil or olive oil when cooking? | 1 point given based on use of perilla oil or olive oil when cooking |
| --- | --- |
| Q2. How much perilla oil or olive oil do you consume per day? | n/a in the KoGES |
| Q3. How many vegetables do you eat per day? | 1 point given based on FFQ calculation, if ≥3 portions of vegetables per day |
| Q4. How many fruits do you eat per day? | 1 point given based on FFQ calculation, if ≥2 portions of fruits per day |
| Q5. How much of red meat (i.e., beef, pork, etc.) and processed meat (ham, sausage, etc.) do you consume per day? | 1 point given based on FFQ calculation, if <2 portions of red meat and processed meats per day |
| Q6. How much butter, margarine, and cream did you consume per day? | 1 point given based on FFQ calculation, if <1 portion of butter and margarine per day |
| Q7. How many drinks that contain sugar do you drink per day (carbonated drinks, juices, processed drinks, etc.)? | 1 point given based on FFQ calculation, if <1 portion of soft drinks per day |
| Q8. How much wine do you consume per week? | 1 point given based on FFQ calculation, if ≥7 cups of wine per week |
| Q9. How much did you consume beans or tofu per week? | 1 point given based on FFQ calculation, if ≥3 portions of beans and tofu per week |
| Q10. How much fish or seafood did you consume per week? | 1 point given based on FFQ calculation, if ≥3 portions of fish and seafood per week |
| Q11. How many times do you consume sweets (chocolate, candy, ice cream, snacks), breads (except whole wheat bread), cakes, and cookies per week? | 1 point given based on FFQ calculation, if <2 times of sweets, cakes, cookies, and breads per week |
| Q12. How many times do you consume nut products per week? | 1 point given based on FFQ calculation, if ≥3 times of nuts per week |
| Q13. Do you have a higher preference to consume white meat (chicken breasts, etc.) rather than red meat (beef, pork, etc.) and processed meat (ham, sausage, etc.)? | 1 point given based on FFQ calculation, if consumption frequency of poultry and chicken > those of red meat and processed meat. |
| Q14. How many times do you consume whole grains (multi-grain rice, rye bread, etc.) per week? | 1 point given based on FFQ calculation, if ≥3 times of whole grains per week |

**Supplementary Table 2. Clinical characteristics of participants without MAFLD based on K-MEDAS based on K-MEDAS scores greater than 6 or less than 5**

|  | Before matching | |  | After matching | |  |
| --- | --- | --- | --- | --- | --- | --- |
| Variables | K-MEDAS>6 | K-MEDAS <5 | P-value | K-MEDAS>6 | K-MEDAS <5 | P-value |
| N | 14484 | 8631 | <0.001 | 446 | 446 |  |
| Age, years | 54.7 ± 7.9 | 52.0 ± 8.4 | <0.001 | 56.1 ± 10.9 | 56.1 ± 10.9 | ns |
| Female, n(%) | 12144 (83.8) | 6089 (70.6) | <0.001 | 232 (52.0) | 232 (52.0) | ns |
| BMI, kg/m2 | 22.8 ± 2.2 | 22.8 ± 2.2 | 0.916 | 22.8 ± 2.2 | 23.0 ± 2.3 | 0.280 |
| WC, cm | 77.1 ± 6.7 | 77.2 ± 6.9 | 0.392 | 79.2 ± 6.8 | 78.1 ± 6.5 | 0.013 |
| SBP, mmHg | 120.3 ± 15.0 | 118.6 ± 15.3 | <0.001 | 118.2 ± 16.2 | 115.7 ± 18.2 | 0.028 |
| DBP, mmHg | 74.2 ± 9.5 | 73.8 ± 10.0 | <0.001 | 74.1 ± 10.3 | 72.6 ± 11.2 | 0.027 |
| FPG, mg/dl | 92.6 ± 16.2 | 91.4 ± 16.3 | <0.001 | 90.8 ± 19.2 | 89.7 ± 18.2 | 0.385 |
| HbA1c, % | 5.6 ± 0.6 | 5.6 ± 0.6 | <0.001 | 5.7 ± 0.8 | 5.6 ± 0.8 | 0.864 |
| Insulin, IU | 7.4 ± 4.5 | 6.8 ± 3.3 | <0.001 | 7.5 ± 7.0 | 6.4 ± 3.0 | 0.017 |
| HOMA-IR | 1.68±1.06 | 1.5±0.83 | <0.001 | 1.62 ± 1.48 | 1.42 ± 0.75 | 0.040 |
| Hs CRP, mg/dl | 0.25±1.1 | 0.25±1.64 | 0.823 | 0.39 ±1.84 | 0.31 ± 0.94 | 0.427 |
| TC, mg/dl | 195.3 ± 35.0 | 194.7 ± 34.0 | 0.196 | 190.0 ± 34.3 | 191.6 ± 33.5 | 0.497 |
| HDL-C, mg/dl | 55.7 ± 13.2 | 55.2 ± 13.0 | 0.001 | 51.6 ± 11.4 | 51.2 ± 11.1 | 0.588 |
| TG, mg/dl | 98.8 ± 47.2 | 96.6 ± 44.5 | <0.001 | 102.8 ± 47.5 | 107.7 ± 45.3 | 0.117 |
| γ-GGT, IU/L | 18.9 ± 14.0 | 19.7 ± 12.8 | <0.001 | 21.6 ± 30.9 | 19.6 ± 11.8 | 0.189 |
| AST, IU/L | 22.5 ± 8.1 | 21.7 ± 8.4 | <0.001 | 22.9 ±10.3 | 23.1 ± 8.1 | 0.780 |
| ALT, IU/L | 19.0 ± 11.3 | 18.3 ± 14.1 | <0.001 | 18.9 ±13.3 | 18.6 ± 8.9 | 0.679 |
| Current drinking, n (%) | 4,354 (30.2) | 3,622 (42.3) | <0.001 | 173 (39.4) | 170 (38.6) | 0.721 |
| Current smoking, n (%) | 515 (3.6) | 917 (10.7) | <0.001 | 82 (18.6) | 74 (16.9) | 0.790 |
| Physical activity, n (%) | 8,654 (59.9) | 3,669 (42.8) | <0.001 | 158 (35.7) | 033 (29.9) | 0.078 |
| Hypertension, n (%) | 2,428 (16.8) | 1,136 (13.2) | <0.001 | 63 (14.2) | 71 (16.0) | 0.521 |
| Type2 diabetes, n (%) | 910 (6.3) | 300 (3.5) | <0.001 | 30 (6.8) | 19 (4.3) | 0.139 |
| Dyslipidemia, n (%) | 1,409 (9.7) | 543 (6.3) | <0.001 | 10 (2.3) | 11 (2.5) | 1.00 |
| Energy, kcal | 1812.9 ± 591.8 | 1595.3 ± 503.3 | <0.001 | 1943.8 ± 667.9 | 1773.1 ± 594.4 | <0.001 |
| Protein, (%) | 13.9 ± 2.6 | 12.5 ± 2.3 | <0.001 | 13.2 ± 2.4 | 13.3 ± 2.4 | 0.475 |
| Fat, (%) | 13.6 ± 5.3 | 13.6 ± 5.5 | <0.001 | 13.5 ± 5.3 | 14.3 ± 5.2 | 0.032 |
| Carbohydrate, (%) | 71.8 ± 6.9 | 72.5 ± 6.8 | <0.001 | 72.3 ± 7.0 | 71.1 ± 6.8 | 0.014 |
| Calcium, mg | 545.9 ± 298.5 | 333.5 ± 190.5 | <0.001 | 504.7 ± 291.7 | 437.6 ± 245.6 | <0.001 |
| Phosphorus, mg | 988.6 ± 393.9 | 749.2 ± 286.4 | <0.001 | 1012.5 ± 415.9 | 934.3 ± 378.5 | 0.004 |
| Iron, mg | 11.7 ± 5.7 | 7.7 ± 3.6 | <0.001 | 11.1 ± 5.5 | 9.7 ± 4.7 | <0.001 |
| Potassium, mg | 2594.7 ± 1201.0 | 1780.7 ± 789.1 | <0.001 | 2563.5 ± 1234.3 | 2226.1 ± 1051.9 | <0.001 |
| Sodium, mg | 2827.3 ±1446.2 | 1882.7 ± 1160.6 | <0.001 | 3164.5 ± 1727.2 | 2687.9 ± 1439.9 | <0.001 |
| Vitamin A, R.E | 575.2 ± 397.9 | 348.1 ± 250.5 | <0.001 | 568.6 ± 441.1 | 466.2 ± 350.4 | <0.001 |
| Vitamin B1, mg | 1.07 ± 0.46 | 0.87 ± 0.39 | <0.001 | 1.2 ± 0.6 | 1.1 ± 0.5 | 0.011 |
| Vitamin B2, mg | 1.0 ± 0.49 | 0.74 ± 0.36 | <0.001 | 0.99 ± 0.49 | 0.9 ± 0.43 | 0.003 |
| Niacin, mg | 15.5 ± 6.7 | 12.3 ± 4.82 | <0.001 | 15.4 ± 6.8 | 14.1 ± 6.0 | 0.002 |
| Vitamin C, mg | 134.6 ± 81.0 | 76.9 ± 52.3 | <0.001 | 134.4 ± 91.2 | 102.2 ± 84.5 | <0.001 |
| Zinc, μg | 8.6 ± 3.8 | 6.73 ± 3.1 | <0.001 | 8.7 ± 3.9 | 8.0 ± 3.8 | 0.008 |
| Vitamin B6, mg | 1.8 ± 0.8 | 1.3 ± 0.5 | <0.001 | 1.8±0.8 | 1.6 ± 0.7 | <0.001 |
| Folate, μg | 261.9 ± 139.0 | 162.9 ± 88.7 | <0.001 | 251.8 ± 133.8 | 215.0 ±112.2 | <0.001 |
| Fiber, g | 7.0 ± 3.3 | 4.3 ± 2.2 | <0.001 | 7.2 ± 3.5 | 6.0 ± 3.2 | <0.001 |
| Vitamin E, mg | 9.3 ± 5.1 | 6.7 ± 3.7 | <0.001 | 9.3 ± 5.6 | 8.1 ± 4.6 | <0.001 |
| Cholesterol, mg | 190.0 ±138.1 | 133.3 ± 97.4 | <0.001 | 170.7 ±133.0 | 165.4 ± 128.6 | 0.552 |

Abbreviations; BMI, Body mass index; WC, Waist circumference; FPG, Fating plasma glucose; Hba1c, glycated hemoglobin; HOMA-IR, Homeostatic Model Assessment for Insulin Resistance; hsCRP, High sensitive C-reactive protein; TC, Total cholesterol; HDL-C, High density lipoprotein cholesterol; TG, Triglyceride; r-GGT, r-glutamyltransferase; AST, aspartate transaminase; ALT, alanine transaminase

**Supplementary Table 3. Clinical characteristics of participants with MAFLD based on K-MEDAS based on K-MEDAS scores greater than 6 or less than 5**

|  | Before matching | |  | After matching | |  |
| --- | --- | --- | --- | --- | --- | --- |
| Variables | K-MEDAS>6 | K-MEDAS <5 | P-value | K-MEDAS>6 | K-MEDAS <5 | P-value |
| N | 5,495 | 4,523 |  | 415 | 415 |  |
| Age, years | 57.0 ± 7.7 | 54.0 ± 8.5 | <0.001 | 55.2 ± 10.3 | 55.2 ±10.3 | ns |
| Female, n(%) | 3277 (59.6) | 1784 (39.4) | <0.001 | 200 (48.2) | 200 (48.2) | ns |
| BMI, kg/m2 | 26.7 ± 2.6 | 26.5 ± 2.5 | 0.001 | 26.7 ± 2.6 | 26.7 ± 2.3 | 0.099 |
| WC, cm | 89.1 ± 6.6 | 88.9 ± 6.3 | 0.115 | 89.4 ± 6.6 | 88.4 ± 6.4 | 0.026 |
| SBP, mmHg | 127.6 ±15.0 | 126.4 ±15.3 | <0.001 | 125.4 ± 15.7 | 122.0 ± 18.8 | 0.004 |
| DBP, mmHg | 78.9 ± 9.7 | 79.0 ± 10.1 | 0.596 | 78.6 ± 10.0 | 76.8 ± 12.3 | 0.021 |
| FPG, mg/dl | 101.3 ± 24.4 | 99.7 ± 24.2 | <0.001 | 98.3 ± 22.5 | 96.3 ±19.1 | 0.165 |
| HbA1c, % | 6.0 ± 0.9 | 5.9 ± 0.9 | 0.015 | 5.9 ± 0.8 | 5.9 ± 0.8 | 0.843 |
| Insulin, IU | 9.6 ± 4.6 | 8.6 ± 3.9 | <0.001 | 8.8 ± 3.8 | 8.3 ± 3.7 | 0.095 |
| HOMA-IR | 2.39 ± 1.89 | 2.09 ± 1.17 | <0.001 | 2.10 ±1.06 | 1.98 ± 1.10 | 0.223 |
| Hs CRP, mg/dl | 0.60 ± 2.47 | 0.44 ± 1.7 | <0.001 | 0.37 ± 0.84 | 0.30 ± 0.65 | 0.244 |
| TC, mg/dl | 203.0 ± 38.2 | 205.4 ± 37.6 | <0.001 | 204.0 ± 33.3 | 210.5 ± 36.5 | 0.007 |
| HDL-C, mg/dl | 46.4 ± 10.6 | 45.8 ± 10.6 | 0.005 | 45.7 ± 10.5 | 45.7 ±10.0 | 0.916 |
| TG, mg/dl | 185.6 ± 107.8 | 190.1 ±110.7 | 0.040 | 193.0 ± 98.6 | 201.8 ± 124.4 | 0.258 |
| γ-GGT, IU/L | 42.0 ± 45.2 | 45.1 ± 41.4 | <0.001 | 42.5 ± 39.8 | 42.9 ± 40.2 | 0.868 |
| AST, IU/L | 27.8 ± 30.0 | 26.5 ± 14.4 | 0.008 | 27.8 ± 17.6 | 27.3 ± 11.6 | 0.637 |
| ALT, IU/L | 29.98±29.58 | 30.36±24.25 | 0.473 | 30.1 ± 27.4 | 29.8 ± 20.9 | 0.855 |
| Current drinking, n (%) | 2,147 (39.2) | 2,331 (51.9) | <0.001 | 183 (44.4) | 185 (44.8) | 0.922 |
| Current smoking, n (%) | 591 (10.8) | 979 (21.8) | <0.001 | 87 (21.0) | 79 (19.1) | 0.716 |
| Physical activity, n (%) | 2,901 (52.9) | 1,944 (43.3) | <0.001 | 172 (41.7) | 142 (34.3) | 0.035 |
| Hypertension, n (%) | 1,864 (34.0) | 380 (8.4) | <0.001 | 107 (25.8) | 109 (26.3) | 0.937 |
| Type2 diabetes, n (%) | 693 (12.6) | 380 (8.4) | <0.001 | 43 (10.4) | 32 (7.7) | 0.230 |
| Dyslipidemia, n (%) | 854 (15.6) | 597 (13.2) | 0.001 | 38 (9.2) | 28 (6.8) | 0.248 |
| Energy, kcal | 1869.2 ± 618.7 | 1659.8 ± 473.0 | <0.001 | 1989.9 ± 827.1 | 1810.4 ± 556.0 | <0.001 |
| Protein, % | 13.8 ± 2.7 | 12.4 ± 2.3 | <0.001 | 13.6 ± 2.5 | 13.4 ± 2.4 | 0.209 |
| Fat, % | 13.2 ± 5.4 | 13.3 ± 5.4 | <0.001 | 13.8 ± 5.6 | 14.1 ± 4.9 | 0.502 |
| Carbohydrate, % | 72.2 ± 7.1 | 72.8 ± 6.8 | <0.001 | 71.7 ± 7.2 | 71.3 ± 6.5 | 0.496 |
| Calcium, mg | 531.2 ± 295.1 | 331.1 ± 183.6 | <0.001 | 514.6 ± 300.0 | 431.0 ± 218.3 | <0.001 |
| Phosphorus, mg | 1003.8 ± 409.5 | 771.8 ± 272.9 | <0.001 | 1041.2 ± 445.3 | 942.2 ± 346.7 | <0.001 |
| Iron, mg | 11.7 ± 5.8 | 8.0 ± 3.4 | <0.001 | 11.7 ± 6.1 | 9.9 ± 4.3 | <0.001 |
| Potassium, mg | 2598.4 ±1212.5 | 1825.3 ± 778.7 | <0.001 | 2657.0 ±1285.7 | 2283.6 ± 980.0 | <0.001 |
| Sodium, mg | 3059.1 ±1587.3 | 2059.0 ±1259.8 | <0.001 | 3316.6 ± 1793.3 | 2815.9 ±1537.5 | <0.001 |
| Vitamin A, R.E | 583.5 ± 416.3 | 366.7 ± 256.9 | <0.001 | 576.6 ± 421.2 | 470.2 ± 306.5 | <0.001 |
| Vitamin B1, mg | 1.11 ± 0.51 | 0.91 ± 0.4 | <0.001 | 1.24 ± 0.71 | 1.12 ± 0.51 | 0.004 |
| Vitamin B2, mg | 1.0 ± 0.51 | 0.76 ± 0.35 | <0.001 | 1.04 ± 0.58 | 0.91 ± 0.42 | <0.001 |
| Niacin, mg | 16.0 ±7.2 | 12.8 ± 4.8 | <0.001 | 16.4 ± 7.9 | 14.5 ± 6.1 | <0.001 |
| Vitamin C, mg | 130.4 ± 78.3 | 77.4 ± 52.4 | <0.001 | 134.0 ± 87.3 | 108.4 ± 74.1 | <0.001 |
| Zinc, μg | 8.8 ± 4.3 | 7.0 ± 3.1 | <0.001 | 8.9 ± 3.9 | 8.1 ± 3.6 | 0.003 |
| Vitamin B6, mg | 1.8 ± 0.8 | 1.4 ± 0.5 | <0.001 | 1.9 ± 0.9 | 1.7 ± 0.7 | <0.001 |
| Folate, μg | 261.0 ±140.6 | 168.7 ± 90.5 | <0.001 | 260.8 ± 141.3 | 220.9 ±107.9 | <0.001 |
| Fiber, g | 7.0 ± 3.3 | 4.5 ± 2.2 | <0.001 | 7.3 ± 3.5 | 6.2 ± 2.9 | <0.001 |
| Vitamin E, mg | 9.3 ± 5.4 | 6.8 ± 3.4 | <0.001 | 10.0 ± 7.5 | 8.2 ± 3.9 | <0.001 |
| Cholesterol, mg | 185.5 ± 142.7 | 132.1 ± 96.1 | <0.001 | 184.3 ±148.8 | 157.3 ± 117.4 | 0.004 |

Abbreviations; BMI, Body mass index; WC, Waist circumference; FPG, Fating plasma glucose; Hba1c, glycated hemoglobin; HOMA-IR, Homeostatic Model Assessment for Insulin Resistance; hsCRP, High sensitive C-reactive protein; TC, Total cholesterol; HDL-C, High density lipoprotein cholesterol; TG, Triglyceride; r-GGT, r-glutamyltransferase; AST, aspartate transaminase; ALT, alanine transaminase

**Supplementary Table 4. Single nucleotide polymorphisms (SNPs) showing significant interactions with K-MEDAS associated with MAFLD**

| SNP | CHR | BP | A1 | gene | ALT | REF | 1000G frequency | | | Total | | K-MEDAS > 6 | | K-MEDAS < 5 | | Genotype x MD | | |
| --- | --- | --- | --- | --- | --- | --- | --- | --- | --- | --- | --- | --- | --- | --- | --- | --- | --- | --- |
|  |  |  |  |  |  |  | EAS | EUR | AMR | OR  (95%CI) | p | OR  (95%CI) | p | OR  (95%CI) | p | OR  (95%CI) | p | |
| rs74439846 | 1 | 245787535 | T | KIF26B | T | G | 0.0734 | 0.0239 | 0.1153 | 1.171(1.09-1.258) | 1.62E-05 | 1.065(0.969-1.171) | 1.93E-01 | 1.337(1.196-1.494) | 3.41E-07 | 0.793(0.678-0.928) | | 3.83E-03 |
| rs6712077 | 2 | 9877600 | C | YWHAQ-TAF1B | T | C | 0.3442 | 0.7097 | 0.732 | 1.053(1.017-1.092) | 4.19E-03 | 1.112(1.062-1.166) | 7.77E-06 | 0.979(0.926-1.035) | 4.50E-01 | 1.126(1.041-1.218) | | 2.96E-03 |
| rs76221744 | 2 | 15528830 | C | NBAS | C | T | 0.0913 | 0 | 0 | 0.89(0.831-0.952) | 7.25E-04 | 0.804(0.734-0.88) | 2.37E-06 | 1.022(0.922-1.134) | 6.76E-01 | 0.726(0.626-0.841) | | 2.08E-05 |
| rs4665972 | 2 | 27598097 | C | EIF2B4 | C | T | 0.5109 | 0.5845 | 0.6427 | 0.888(0.857-0.92) | 9.15E-11 | 0.847(0.808-0.888) | 5.05E-12 | 0.944(0.893-0.998) | 4.37E-02 | 0.894(0.826-0.967) | | 5.22E-03 |
| rs750801245 | 2 | 27674931 | A | IFT172 | A | AAAC | 0.3194 | 0.285 | 0.108 | 0.893(0.859-0.929) | 1.19E-08 | 0.873(0.829-0.918) | 1.75E-07 | 0.919(0.865-0.976) | 5.73E-03 | 0.907(0.833-0.988) | | 2.50E-02 |
| rs780094 | 2 | 27741237 | C | GCKR | C | T | 0.5238 | 0.5895 | 0.6398 | 0.877(0.847-0.909) | 5.54E-13 | 0.843(0.804-0.883) | 5.80E-13 | 0.925(0.875-0.978) | 5.72E-03 | 0.911(0.843-0.985) | | 1.93E-02 |
| rs147530106 | 2 | 195052976 | T | PCGEM1-LOC101927406 | T | C | 0.0119 | 0.001 | 0.0072 | 1.322(1.145-1.527) | 1.47E-04 | 1.527(1.274-1.831) | 4.74E-06 | 1.059(0.836-1.34) | 6.36E-01 | 1.523(1.101-2.107) | | 1.11E-02 |
| rs34872 | 3 | 10508495 | T | ATP2B2 | C | T | 0.2629 | 0.4791 | 0.4135 | 1.069(1.03-1.109) | 4.59E-04 | 1.133(1.079-1.189) | 4.85E-07 | 0.99(0.934-1.049) | 7.28E-01 | 1.121(1.033-1.218) | | 6.44E-03 |
| rs2280305 | 3 | 13660809 | C | SNORA93 | C | T | 0.3601 | 0.1103 | 0.1744 | 1.08(1.039-1.122) | 1.10E-04 | 1.03(0.979-1.084) | 2.53E-01 | 1.155(1.087-1.227) | 3.30E-06 | 0.891(0.818-0.97) | | 7.49E-03 |
| rs9842140 | 3 | 134192469 | C | ANAPC13 | A | C | 0.4425 | 0.6233 | 0.4654 | 1.071(1.033-1.111) | 1.98E-04 | 1.118(1.066-1.172) | 4.45E-06 | 1.009(0.954-1.067) | 7.52E-01 | 1.109(1.024-1.2) | | 1.07E-02 |
| rs41487049 | 3 | 150717828 | T | CLRN1-AS1 | T | C | 0.0843 | 0.1332 | 0.0836 | 1.079(1.007-1.156) | 3.19E-02 | 0.933(0.849-1.024) | 1.44E-01 | 1.295(1.167-1.438) | 1.22E-06 | 0.69(0.593-0.804) | | 1.76E-06 |
| rs61798175 | 3 | 178740425 | T | ZMAT3 | A | T | 0.3889 | 0.2913 | 0.2493 | 0.948(0.915-0.983) | 3.37E-03 | 0.883(0.843-0.926) | 2.18E-07 | 1.048(0.992-1.108) | 9.54E-02 | 0.862(0.797-0.932) | | 2.04E-04 |
| rs10013804 | 4 | 57647319 | G | HOPX-SPINK2 | A | G | 0.0615 | 0.4046 | 0.3991 | 0.847(0.785-0.913) | 1.56E-05 | 0.929(0.843-1.024) | 1.39E-01 | 0.738(0.655-0.831) | 5.98E-07 | 1.285(1.088-1.518) | | 3.10E-03 |
| rs118027587 | 4 | 170346528 | A | NEK1 | A | G | 0.0883 | 0.0129 | 0.0937 | 0.913(0.86-0.97) | 2.99E-03 | 0.829(0.765-0.898) | 4.14E-06 | 1.036(0.946-1.135) | 4.47E-01 | 0.816(0.716-0.93) | | 2.30E-03 |
| rs77270315 | 5 | 87520214 | T | TMEM161B | T | C | 0.0228 | 0.001 | 0.0014 | 1.158(1.063-1.261) | 7.34E-04 | 1.29(1.156-1.439) | 5.25E-06 | 0.989(0.864-1.132) | 8.70E-01 | 1.289(1.067-1.558) | | 8.47E-03 |
| rs117241353 | 6 | 64258419 | T | LGSN-PTP4A1 | T | A | 0.0496 | 0 | 0 | 1.183(1.064-1.316) | 1.91E-03 | 1.409(1.229-1.616) | 8.89E-07 | 0.926(0.783-1.095) | 3.69E-01 | 1.547(1.222-1.959) | | 2.92E-04 |
| rs58534189 | 6 | 76231982 | C | FILIP1-SENP6 | C | T | 0.2887 | 0.4791 | 0.3386 | 0.925(0.886-0.965) | 2.76E-04 | 0.879(0.831-0.929) | 5.59E-06 | 0.99(0.928-1.057) | 7.72E-01 | 0.87(0.793-0.954) | | 3.22E-03 |
| rs142276542 | 6 | 112835656 | T | RFPL4B-LOC101927686 | T | G | 0.0188 | 0 | 0 | 1.181(1.042-1.339) | 9.35E-03 | 1.443(1.23-1.692) | 6.79E-06 | 0.869(0.71-1.063) | 1.73E-01 | 1.749(1.322-2.313) | | 8.96E-05 |
| rs1123164 | 7 | 1261046 | T | LOC101927021-UNCX | T | C | 0.3304 | 0.4692 | 0.4914 | 1.041(1.004-1.079) | 2.87E-02 | 1.117(1.066-1.171) | 3.86E-06 | 0.944(0.892-0.998) | 4.31E-02 | 1.148(1.06-1.242) | | 6.83E-04 |
| rs1352659358 | 7 | 1269592 | AC | UNCX | A | AC | 0.4048 | 0.1779 | 0.272 | 0.939(0.904-0.976) | 1.45E-03 | 0.891(0.847-0.938) | 9.05E-06 | 1.006(0.948-1.067) | 8.56E-01 | 0.903(0.83-0.983) | | 1.80E-02 |
| rs1261608997 | 7 | 69553513 | C | AUTS2 | C | CT | 0.3353 | 0.3022 | 0.31 | 1.078(1.038-1.12) | 1.17E-04 | 1.036(0.985-1.089) | 1.66E-01 | 1.144(1.078-1.214) | 9.92E-06 | 0.881(0.81-0.958) | | 3.14E-03 |
| rs147137343 | 7 | 84027732 | T | SEMA3A-LOC101927378 | T | A | 0.005 | 0 | 0 | 1.223(1.033-1.449) | 1.93E-02 | 0.896(0.706-1.138) | 3.68E-01 | 1.77(1.376-2.276) | 8.78E-06 | 0.538(0.37-0.783) | | 1.20E-03 |
| rs182367731 | 7 | 84741071 | A | SEMA3D | A | G | 0.005 | 0 | 0 | 1.156(0.977-1.368) | 9.19E-02 | 0.814(0.641-1.033) | 9.08E-02 | 1.772(1.379-2.278) | 7.97E-06 | 0.496(0.341-0.722) | | 2.48E-04 |
| rs1242783 | 7 | 157498768 | A | PTPRN2 | A | G | 0.5069 | 0.173 | 0.3401 | 1.07(1.032-1.109) | 2.04E-04 | 1.114(1.063-1.167) | 5.57E-06 | 1.011(0.957-1.069) | 6.92E-01 | 1.096(1.014-1.185) | | 2.14E-02 |
| rs2642369 | 8 | 104302410 | T | BAALC-FZD6 | T | A | 0.0734 | 0.3052 | 0.1671 | 0.894(0.84-0.951) | 3.70E-04 | 0.965(0.89-1.045) | 3.78E-01 | 0.798(0.725-0.88) | 5.13E-06 | 1.265(1.104-1.45) | | 7.16E-04 |
| rs827526 | 8 | 104311523 | A | FZD6 | A | C | 0.0724 | 0.3072 | 0.1888 | 0.894(0.841-0.951) | 3.86E-04 | 0.965(0.891-1.045) | 3.81E-01 | 0.8(0.726-0.881) | 5.51E-06 | 1.263(1.103-1.446) | | 7.52E-04 |
| rs116950707 | 9 | 1613507 | T | DMRT2-SMARCA2 | T | C | 0.0298 | 0 | 0.0202 | 1.149(1.073-1.23) | 7.51E-05 | 1.235(1.131-1.349) | 2.40E-06 | 1.041(0.933-1.162) | 4.74E-01 | 1.168(1.002-1.361) | | 4.68E-02 |
| rs4838333 | 9 | 128842790 | T | PBX3-LOC101929116 | T | C | 0.0575 | 0.2535 | 0.2954 | 0.89(0.829-0.955) | 1.22E-03 | 0.786(0.715-0.864) | 5.84E-07 | 1.06(0.951-1.182) | 2.91E-01 | 0.767(0.656-0.898) | | 9.42E-04 |
| rs985957602 | 10 | 59697844 | A | MIR3924-IPMK | A | AAT | 0.0526 | 0.0487 | 0.037 | 1.193(1.085-1.311) | 2.72E-04 | 1.319(1.168-1.489) | 7.82E-06 | 1.036(0.891-1.206) | 6.44E-01 | 1.325(1.07-1.64) | | 9.80E-03 |
| rs1945440 | 11 | 21423652 | G | NELL1 | A | G | 0.3859 | 0.3598 | 0.3746 | 0.952(0.918-0.988) | 9.46E-03 | 1.016(0.968-1.066) | 5.21E-01 | 0.872(0.823-0.924) | 3.26E-06 | 1.148(1.059-1.244) | | 8.44E-04 |
| rs12292238 | 11 | 78823774 | C | TENM4 | C | T | 0.0466 | 0.175 | 0.0965 | 0.873(0.804-0.947) | 1.11E-03 | 0.775(0.693-0.865) | 6.06E-06 | 1.013(0.895-1.146) | 8.43E-01 | 0.751(0.629-0.896) | | 1.48E-03 |
| rs11223302 | 11 | 132834193 | C | OPCML | C | A | 0.0754 | 0.0686 | 0.0346 | 1.115(1.037-1.199) | 3.27E-03 | 1.004(0.911-1.106) | 9.38E-01 | 1.291(1.155-1.442) | 6.70E-06 | 0.769(0.655-0.901) | | 1.21E-03 |
| rs1223643480 | 12 | 77465743 | ATTTT | E2F7-NAV3 | A | ATTTT | 0.381 | 0.841 | 0.769 | 0.941(0.908-0.976) | 1.05E-03 | 0.898(0.856-0.942) | 9.51E-06 | 1.005(0.951-1.062) | 8.69E-01 | 0.896(0.828-0.97) | | 6.45E-03 |
| rs151297276 | 12 | 108678536 | A | CMKLR1 | A | G | 0.0308 | 0 | 0.0562 | 1.099(1.009-1.197) | 2.96E-02 | 1.292(1.155-1.446) | 7.46E-06 | 0.886(0.777-1.01) | 6.92E-02 | 1.424(1.182-1.715) | | 1.99E-04 |
| rs9533638 | 13 | 44401002 | A | ENOX1-CCDC122 | A | G | 0.253 | 0.166 | 0.2262 | 1.073(1.032-1.116) | 3.63E-04 | 1.129(1.073-1.188) | 2.85E-06 | 0.997(0.938-1.059) | 9.15E-01 | 1.122(1.03-1.222) | | 8.50E-03 |
| rs148402789 | 13 | 72983163 | T | DACH1-MZT1 | T | G | 0.0099 | 0.001 | 0 | 1.246(1.098-1.413) | 6.29E-04 | 1.043(0.88-1.236) | 6.31E-01 | 1.569(1.295-1.901) | 4.24E-06 | 0.665(0.504-0.877) | | 3.84E-03 |
| rs58293364 | 13 | 111066596 | A | COL4A2 | A | G | 0.0179 | 0.006 | 0.0014 | 1.332(1.162-1.526) | 3.81E-05 | 1.105(0.921-1.326) | 2.82E-01 | 1.739(1.408-2.148) | 2.89E-07 | 0.71(0.523-0.963) | | 2.76E-02 |
| rs4430854 | 18 | 63990744 | G | CDH7-CDH19 | A | G | 0.4464 | 0.3777 | 0.4625 | 1.058(1.021-1.096) | 2.02E-03 | 1.003(0.957-1.051) | 8.99E-01 | 1.137(1.076-1.201) | 5.31E-06 | 0.889(0.822-0.962) | | 3.23E-03 |
| rs12973650 | 19 | 20734253 | G | ZNF737 | G | A | 0.4276 | 0.4215 | 0.4914 | 1.061(1.024-1.099) | 1.10E-03 | 1.11(1.06-1.162) | 9.65E-06 | 0.995(0.942-1.051) | 8.58E-01 | 1.121(1.038-1.211) | | 3.75E-03 |
| rs6128763 | 20 | 58709709 | T | MIR646HG | C | T | 0.2272 | 0.5686 | 0.3862 | 0.923(0.882-0.965) | 4.25E-04 | 0.867(0.817-0.92) | 2.68E-06 | 1.004(0.937-1.075) | 9.19E-01 | 0.852(0.773-0.94) | | 1.38E-03 |

**Supplementary Table 5. Single nucleotide polymorphisms (SNPs) showing significant interactions with K-MEDAS associated with MASLD according to sex**

| **SNP** | **CHR** | **BP** | **A1** | | **gene** | **ALT** | | **REF** | | **EAS** | | **EUR** | **AMR** |
| --- | --- | --- | --- | --- | --- | --- | --- | --- | --- | --- | --- | --- | --- |
| rs780094 | 2 | 27741237 | C | | GCKR | C | | T | | 0.5238 | | 0.5895 | 0.6398 |
| SNP | Male | | | | | | | | | | | | |
|  | Total  (case: 4,957/con: 4,882) | | | K-MEDAS >6  (case: 2,218/con:2,340) | | | K-MEDAS <5  (case: 2,739/con:2,542) | | | |  | | |
| rs780094 | OR (95% CI) | p-value | | OR (95% CI) | p-value | | OR (95% CI) | | p-value | |  | |  |
|  | 0.87 (0.82-0.92) | 1.6e^-06^ | | 0.80 (0.74-0.88) | 4.4e^-07^ | | 0.93 (0.86-1.01) | | 6.9e^-02^ | |  | |  |
|  | Female | | | | | | | | | | | | |
|  | Total  (case: 5,061/con:18,233) | | | K-MEDAS >6  (case:3,277/con:12,144) | | | K-MEDAS <5  (case: 1,784/con:6,089) | | | |  | | |
|  | OR (95% CI) | p-value | | OR (95% CI) | p-value | | OR (95% CI) | | p-value | |  | |  |
|  | 0.88 (0.84-0.92) | 2.6e^-08^ | | 0.86 (0.81-0.91) | 6.7e^-08^ | | 0.92 (0.85-0.99) | | 3.1e^-02^ | |  | |  |
